# The diagnostic performance of ultrasound elastography for biliary atresia: A meta-analysis

**DOI:** 10.3389/fpubh.2022.973125

**Published:** 2022-10-26

**Authors:** Bingtian Dong, Zongjie Weng, Guorong Lyu, Xiaocen Yang, Huaming Wang

**Affiliations:** ^1^Department of Ultrasound, The Second Affiliated Hospital of Fujian Medical University, Quanzhou, China; ^2^Department of Medical Ultrasonics, Fujian Provincial Maternity and Children's Hospital, Affiliated Hospital of Fujian Medical University, Fuzhou, China; ^3^Collaborative Innovation Center for Maternal and Infant Health Service Application Technology, Quanzhou Medical College, Quanzhou, China; ^4^Department of Ultrasound, Chenggong Hospital, Xiamen University, Xiamen, China

**Keywords:** ultrasound elastography, biliary atresia, diagnosis, stiffness, meta-analysis

## Abstract

**Background:**

Biliary atresia (BA) is a severe inflammatory obliterative cholangiopathy of infancy that requires early diagnosis and prompt surgical intervention. In this study, we aimed to obtain comprehensive evidence on the diagnostic performance of liver stiffness measurement by ultrasound elastography in the detection of BA through a meta-analysis.

**Methods:**

The PubMed, EMBASE, Cochrane Library, and Web of Science databases were searched for studies that investigated the diagnostic performance of ultrasound elastography in the detection of BA up to January 10, 2022. In this study, in order to summarize the diagnostic performance of ultrasound elastography, the summary receiver operating characteristic (SROC) modeling was constructed. Heterogeneity was estimated with the *I*^2^ statistic. Multiple subgroup analyses were also performed.

**Results:**

Fourteen studies from eleven articles, including 774 BA patients, 850 non-BA patients, and 173 controls were included in the present meta-analysis. The summary sensitivity and specificity of ultrasound elastography for liver stiffness were 85% [95% confidence interval (CI): 79–89%] and 82% (95% CI: 73–88%) with the *I*^2^ value of 82.90 and 84.33%, respectively. The area under the SROC curve (AUROC) using ultrasound elastography for diagnosing BA was 0.90 (95% CI: 0.87–0.92). In addition, a subgroup analysis of 9 two-dimensional shear wave elastography studies was also performed. Subgroup analysis revealed that the summary sensitivity and specificity were 85% (95% CI: 77–91%) and 79% (95% CI: 71–86%), respectively, and the summary AUROC was 0.89 (95% CI: 0.86–0.92).

**Conclusions:**

Ultrasound elastography exhibits good diagnostic accuracy for BA and can be served as a non-invasive tool to facilitate the differential diagnosis of BA.

## Introduction

Biliary atresia (BA) is a severe inflammatory obliterative cholangiopathy of infancy (1, 2). This disease is a global problem, and its incidence rate varies markedly across different regions (3–7). If not treated timely, BA would eventually progress into end-stage liver disease, and finally leading to death in the first 2 years of life (8). A surgical procedure called Kasai portoenterostomy (KPE) is the current treatment option (1, 9). In fact, the success of KPE surgery for BA depends in large part on the age at which it is performed (8). Therefore, early diagnosis plays a vital role.

However, in infants with cholestasis, early identification of BA remains challenging (10, 11). Currently, several modalities have been chosen to evaluate the potential anomaly of biliary system, such as conventional ultrasonography, hepatobiliary scintigraphy, and magnetic resonance cholangiopancreatography (MRCP) (12, 13). Unfortunately, both hepatobiliary scintigraphy and MRCP provided a relatively low specificity for diagnosis of BA (14–16). The ultrasonographic features of gallbladder abnormalities are suggestive of BA, but require an analyst with expertise (10, 17). Although liver biopsy or intraoperative cholangiography (IOC) has traditionally been regarded as a relatively accurate test for the diagnosis of BA, it is an invasive procedure (12, 18).

Recently, ultrasound elastography has been developed as a novel quantitative sonographic technique to assist in this effort through the non-invasive measurement of liver stiffness (2). Transient elastography (TE) is the most widely validated shear wave-based elastography technique (19, 20). Nevertheless, it has certain drawbacks (20–23). Several studies using this technique have shown that it has more technical failures in young children (24, 25). Point shear wave elastography (p-SWE) and two-dimensional shear wave elastography (2D-SWE) are recently developed techniques (19). Importantly, 2D-SWE is able to directly visualize the elasticity measurements by displaying a color coding elastographic map on a gray-scale ultrasound image in real time (21, 26). Previous research has already demonstrated that 2D-SWE is an efficient tool for the diagnosis of liver fibrosis, comparing favorably to TE and p-SWE (27).

To date, there are accumulating reports on the value of ultrasound elastography in the diagnosis of BA; however, they utilized relatively small samples. Moreover, there is variability in the diagnostic performance presented in previous studies. In this study, therefore, we aimed to obtain comprehensive evidence on the diagnostic performance of ultrasound elastography in BA through a meta-analysis.

## Methods

This meta-analysis followed the Preferred Reporting Items for Systematic Reviews and Meta-Analyses (PRISMA) guidelines (28). Within this paper, we focus on analyzing the diagnostic performance of ultrasound elastography for BA.

### Literature search strategy

A computerized search was conducted using the PubMed, EMBASE, Cochrane Library, and Web of Science databases to identify studies, which evaluated the diagnostic value of ultrasound elastography for BA up to January 10, 2022. The search terms included biliary atresia, atresia, extrahepatic biliary atresia, biliary, elastography and stiffness. In addition, we also examined the references of the initially identified articles to identify additional relevant publications. All initial records were exported to Endnote (version X9).

### Inclusion and exclusion criteria

Studies were included if they fulfilled following criteria: (1) the study evaluated the accuracy of liver stiffness measurement by ultrasound elastography for the diagnosis of BA; (2) the study enrolled more than 10 infants with BA; and (3) the study had sufficient data, thus allowing us to construct 2 **×** 2 contingency tables for test performance for further analysis. Studies meeting any of the following criteria were excluded: (1) studies were not relevant to ultrasound elastography diagnosis; (2) non-original research articles, including reviews, conference abstracts, letters, protocols, guidelines and commentary; (3) data incomplete; and (4) studies published in non-English language.

### Data extraction

Using a standardized form, the following data were extracted from the selected eligible studies: (1) study characteristics, such as first author, year of publication, and region; (2) demographic and clinical characteristics, such as age, male/female ratio, and reference standard; and (3) technical characteristics of ultrasound elastography, such as ultrasound elastography system, type of ultrasound elastography, and probe. In addition, a 2 **×** 2 contingency table was also builded using the data retrieved from each study. If one article has evaluated more than one type of ultrasound elastography methods, we considered each type of ultrasound elastography method as an independent study.

The revised Quality Assessment of Diagnostic Accuracy Studies-2 (QUADAS-2) tool was used to assess the quality of the studies included in this analysis (29). Two researchers independently searched the databases, read and selected the articles to include in the present meta-analysis, extracted the required information from the included studies, and assessed the quality of the included studies. If there were disagreements between the two authors, a third review author would be consulted.

### Data synthesis and analysis

To assess the diagnostic performance of ultrasound elastography for the detection of BA, the summary sensitivity, specificity and the corresponding 95% confidence intervals (CIs) were calculated using a random-effect model in the present meta-analysis. In addition, using the data from different studies, we also simultaneously constructed the summary receiver operating characteristic (SROC) curves of ultrasound elastography in diagnosing BA. Three methods, including the summary sensitivities and specificities, the summary diagnostic odds ratios (DORs) and area under the SROC curve (AUROC), were used to examine the accuracy of ultrasound elastography for the detection of BA.

Heterogeneity was assessed using the Cochrane-*Q* test and the inconsistency index *I*^2^ statistic (30). *I*^2^ value > 50% is suggestive of substantial heterogeneity. Spearman correlation coefficient was also calculated to evaluate the threshold effect. Subgroup analysis and meta-regression analysis were performed to explore the potential source of heterogeneity. In this meta-analysis, a subgroup analysis was also conducted to assess the performance of the relatively new type of ultrasound elastography (2D-SWE) for the diagnosis of BA.

We used the Deeks' funnel plot asymmetry test to evaluate possible publication bias (31). A *P* < 0.05 was considered to indicate a significant bias. In the present meta-analysis, the analyses were conducted using Stata version 15.0 (Stata Corp.).

## Results

### Literature search

**Figure 1:**
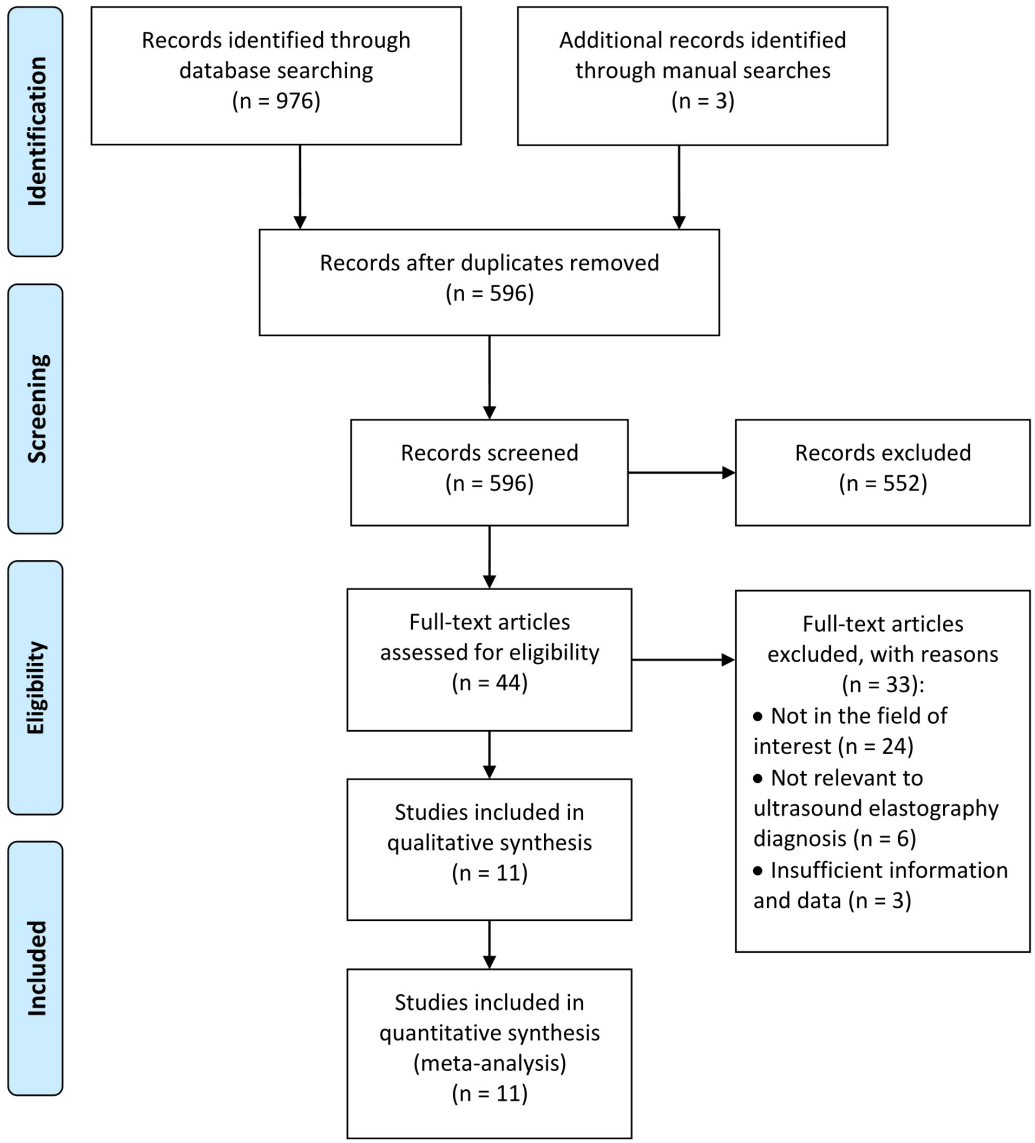
Flow diagram of the study.

Figure 1 displays the flow diagram. Initially, our search strategy retrieved 979 records. A total of 596 records were then retained after removing duplications. Furthermore, 585 studies were excluded, including reviews, conference abstracts, protocols, studies not relevant to ultrasound elastography diagnosis, or studies with insufficient information and data, etc. Finally, 11 studies (12, 32–41) were included in this meta-analysis.

### Study and technical characteristics

Basic characteristics of the included studies are summarized in Table 1. Totally, 14 studies (nine 2D-SWE studies, three p-SWE studies, and two TE studies) from 11 articles were identified for evaluation. All studies were published between 2016 and 2022, of which, six studies were published between 2020 and 2022. Most of the studies included in the present meta-analysis were from China. Overall, 1,797 subjects, including 774 BA patients, 850 non-BA (i.e., other causes of cholestasis) patients, and 173 controls, were included in this meta-analysis. Patients in the BA group had a mean age of 50.2 days, and ~47.0% were male (range: 30.8–60.0%). Methodological quality of included studies according to QUADAS-2 is displayed in Supplementary material.

**Table 1:** Basic characteristics of the included studies.

**References**	**Region**	**Study design**	**Study period**	**Group**	**Size**	**Mean/median age, days**	**Age range, days**	**Male/female**
Boo et al. (12)	Taiwan	Prospective	Jan 2018–Aug 2019	BA	15	30	22–63 (IQR)	9/6
				Non-BA	46	35.5	24–51.3 (IQR)	34/12
Wang et al. (32)	China	NA	Mar 2014–Apr 2015	BA	38	42	16 days−5 months	14/24
				Non-BA	17	50	16 days−5 months	10/7
				Control	31	65	1 month−5 months	18/13
Zhou et al. (33)	China	Prospective	Jan 2012–May 2015	BA	97	65.3	26–134	55/42
				Non-BA	75	62.4	2–140	57/18
				Control	40	39.1	1–160	23/17
Wu et al. (34)	Taiwan	Prospective	May 2015–Dec 2017	BA	15	45	34.5–60.5 (IQR)	7/8
				Non-BA	33	40	27–56 (IQR)	24/9
Dillman et al. (35)	USA	Prospective	Sep 2016–Dec 2018	BA	13	37.0	30.8–56.3 (IQR)	4/9
				Non-BA	28	31.5	18.5–51.5 (IQR)	8/20
Duan et al. (36)	China	NA	Nov 2016–Dec 2017	BA	51	43	5–88	18/33
				Non-BA	87	30	5–90	58/29
				Control	62	35	7–90	28/34
Shen et al. (37)	China	Retrospective	Jan 2016–Dec 2018	BA	135	59	±18.8 (SD)	65/70
				Non-BA	147	70	±20.4 (SD)	106/41
Liu et al. (38)	China	NA	NA	BA	26	72.5	30–127	15/11
				Non-BA	33	81.3	25–141	16/17
				Control	40	71.9	13–150	24/16
Sandberg et al. (39)	USA	Prospective	Nov 2017–Nov 2019	BA	212	59.7	20–114	99/113
				Non-BA	106	65.7	9–186	76/30
Wang et al. (40)	China	Prospective	Jan 2018–Oct 2019	BA	89	46/50^a^	33–54/33–57 (IQR)^a^	41/48
				Non-BA	205	47/44^a^	36–53/34–52 (IQR)^a^	146/59
Liu et al. (41)	China	Retrospective	Feb 2016–Dec 2020	BA	83	NA	NA	43/40
				Non-BA	73	NA	NA	50/23

BA, biliary atresia; IQR, interquartile range; NA, not available; Non-BA, not biliary atresia; SD, standard deviation.

a Data for training cohort and validation cohort, respectively.

Technical characteristics of ultrasound elastography in the included studies are given in Table 2. For the measurement of liver stiffness, a total of three types of ultrasound elastography techniques (i.e., TE, p-SWE, and 2D-SWE) were used in the included studies. Specifically, two studies were performed with TE, three studies involved both p-SWE and 2D-SWE, and six studies were performed with 2D-SWE only. Among the included studies, the devices used to perform ultrasound elastography varied, including Acuson in six studies, Aixplorer in five studies, FibroScan 502 Touch in two studies, and TUS-Aplio 500 in one study. On the other hand, based on the technique, ultrasound elastography can be categorized as TE (FibroScan 502 Touch; Echosens), p-SWE (Virtual Touch Quantification; Siemens Healthineers), and 2D-SWE, including ShearWave Elastography (SuperSonic Imagine), Virtual Touch Tissue and Imaging Quantification (Siemens Healthineers) and Acoustic Structure Quantification (Toshiba Medical Systems). With regard to the measure of liver stiffness, eight ultrasound elastography studies (two TE studies and six 2D-SWE studies) used elasticity (in kilopascals) and six studies (three p-SWE studies and three 2D-SWE studies) used shear wave speed (in meters per second). We summarized the liver stiffness measurement by ultrasound elastography of infants in different groups in the included studies, as illustrated in Table 3.

**Table 2:** Characteristics of ultrasound elastography measurements.

**References**	**Technique**	**US elastography systems**	**Probe**	**No. of measurements**	**No. of readers**	**Reader blinding**	**Reference standard**
Boo et al. (12)	TE	FibroScan 502^a^	S1 (5 MHz)	10	NA	NA	IOC and liver transplantation
Wang et al. (32)	2D-SWE	Aixplorer^b^	L15-4	NA	NA	NA	Pathologic examination
Zhou et al. (33)	2D-SWE	Aixplorer^b^	SL15-4	3	1	Yes	Surgical exploration, IOC and liver biopsy
Wu et al. (34)	TE	FibroScan 502^a^	S1 (5 MHz)	10	NA	Yes	IOC
Dillman et al. (35)	p-SWE	Virtual Touch Q^c^	9L4	10	NA	NA	Clinically suspected BA
	2D-SWE	Virtual Touch Imaging Q^c^	9L4	8	NA	NA	Clinically suspected BA
Duan et al. (36)	2D-SWE	TUS-Aplio 500^d^	14L5 (10 MHz)	5	1	NA	Surgery and pathological findings
Shen et al. (37)	2D-SWE	Aixplorer^b^	L15-4	5	2	NA	Surgery
Liu et al. (38)	p-SWE	Virtual Touch Q^c^	6C1 (3–5.5 MHz)	6	2	NA	Exploratory laparotomy
	2D-SWE	Virtual Touch Imaging Q^c^	9L4 (4-9 MHz)	5-7	2	NA	Exploratory laparotomy
Sandberg et al. (39)	p-SWE	Virtual Touch Q^c^	C6, L9	≥ 5	NA	NA	Tissue biopsy
	2D-SWE	Virtual Touch Imaging Q^c^	L9	≥ 5	NA	NA	Tissue biopsy
Wang et al. (40)	2D-SWE	Aixplorer^b^	Linear probe	6	1	Yes	IOC and liver biopsy
Liu et al. (41)	2D-SWE	Aixplorer^b^	L15-4	6	1	NA	IOC and histological examination

BA, biliary atresia; IOC, intraoperative cholangiography; NA, not available; p-SWE, point shear wave elastography; ROI, region of interest; TE, transient elastography; 2D-SWE, two-dimensional shear wave elastography; US, ultrasound.

a Echosense.

b SuperSonic Imagine.

c Siemens Healthineers.

d Toshiba Medical Systems.

**Table 3:** The LSM of infants in different groups in the included studies.

**References**	**Technique**	**Representative values**	**BA group**			**Non-BA group**			**Control group**		
			***n***	**LSM**	**Range**	***n***	**LSM**	**Range**	***n***	**LSM**	**Range**
Boo et al. (12)	TE	Median	15	13 kPa	7.8–19.7 (IQR)	46	4.9 kPa	3.6–5.7 (IQR)	–	–	–
Wang et al. (32)	2D-SWE	Mean	38	20.46 kPa	±10.19	17	6.29 kPa	±0.99	31	6.41 kPa	±1.08
Zhou et al. (33)	2D-SWE	Mean	97	12.6 kPa (median)	10.6–18.8 (IQR)	75	9.6 kPa (median)	7.5–11.7 (IQR)	40	5.5 kPa	3.7–7.7
Wu et al. (34)	TE	Median	15	10.5 kPa	8.5–20.9 (IQR)	33	4.6 kPa	3.9–6.0 (IQR)	–	–	–
Dillman et al. (35)	p-SWE	Median	13	1.95 m/s	1.48–2.42 (IQR)	28	1.21 m/s	1.12–1.51 (IQR)	–	–	–
	2D-SWE	Median	13	2.08 m/s	1.90–2.50 (IQR)	28	1.49 m/s	1.34–1.80 (IQR)	–	–	–
Duan et al. (36)	2D-SWE	Mean	51	17.72 kPa	±5.89	87	9.88 kPa	±1.84	62	6.63 kPa	±0.54
Shen et al. (37)	2D-SWE	Mean	135	12 kPa (median)	6.0 (IQR)	147	8.1 kPa (median)	3.3 (IQR)	–	–	–
Liu et al. (38)	p-SWE	Mean	26	2.36 m/s	±0.36	33	1.30 m/s	±0.28	40	1.09 m/s	±0.18
	2D-SWE	Mean	26	2.43 m/s	±0.29	33	1.52 m/s	±0.29	40	1.36 m/s	±0.21
Sandberg et al. (39)	p-SWE^a^	Median	212	2.1 m/s	1.7–2.4 (IQR)	106	1.5 m/s	1.3–1.9 (IQR)	–	–	–
	2D-SWE	Median	212	2.2 m/s	1.9–2.5 (IQR)	106	1.8 m/s	1.6–2.1 (IQR)	–	–	–
Wang et al. (40)	2D-SWE	Median	43/46^b^	9.9/11.1 kPa^b^	8.4–14.3/8.7–12.8 (IQR)^b^	107/98^b^	6.6/6.3 kPa^b^	5.7–7.5/5.3–7.7 (IQR)^b^	–	–	–
Liu et al. (41)	2D-SWE	Median	83	9.37 kPa	7.30–11.45 (IQR)	73	6.5 kPa	5.95–7.65 (IQR)	–	–	–

BA, biliary atresia; IQR, interquartile range; LSM, liver stiffness measurement; Non-BA, not biliary atresia; p-SWE, point shear wave elastography; SD, standard deviation; TE, transient elastography; 2D-SWE, two-dimensional shear wave elastography.

a Data were obtained using L9 point shear wave elastography (p-SWE).

b Data for training cohort and validation cohort, respectively.

### Diagnostic performance

Fourteen studies from eleven articles provided information regarding the performance of liver stiffness measurement by ultrasound elastography for diagnosing BA.

**Figure 2:**
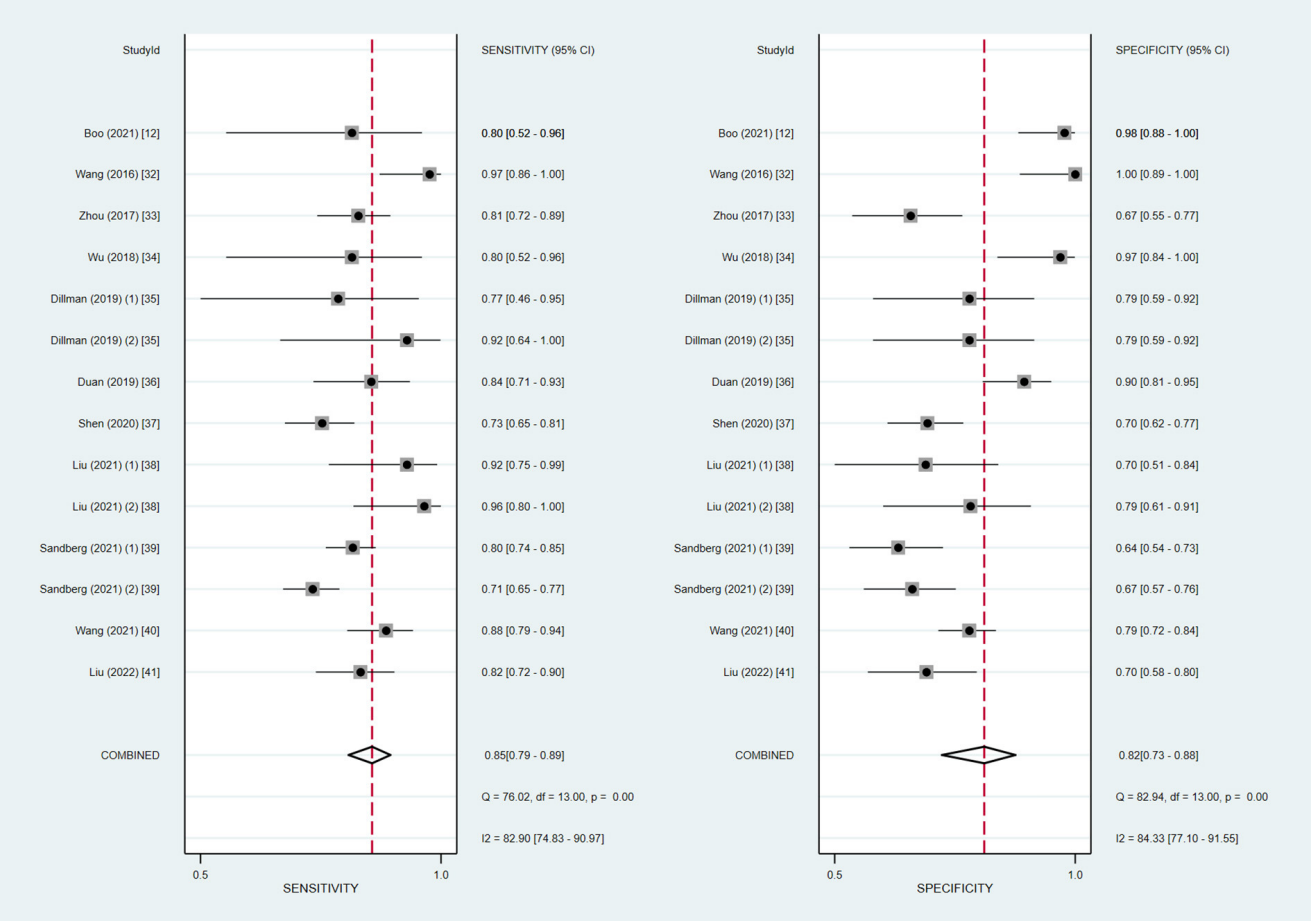
Coupled forest plots of the summary sensitivity and specificity of ultrasound elastography for the diagnosis of biliary atresia (BA).

We extracted the raw data for diagnostic test accuracy, and then these were used to examine the accuracy of ultrasound elastography for the diagnosis of BA (Table 4). Among the 14 included studies, the sensitivity and specificity ranged from 71 to 97.4% and 64 to 100%, respectively. Based on the combined results, the summary sensitivity and specificity of liver stiffness measurement by ultrasound elastography for the prediction of BA were 85% (95% CI: 79–89%) and 82% (95% CI: 73–88%) with the *I*^2^ value of 82.90 and 84.33%, respectively (Figure 2). The cut-off values from each study are displayed in Table 4. Liver stiffness was measured using a unit of elasticity (in kilopascals) in the two TE studies with a cut-off value of 7.7 kPa for each study, and in the six 2D-SWE studies with a mean cut-off value of 9.3 kPa (range: 7.10–12.35 kPa). In addition, the three p-SWE studies and the other three 2D-SWE studies used a unit of shear wave speed (in meters per second), with mean cut-off values of 1.6 m/s (range: 1.53–1.77 m/s) and 1.92 m/s (range: 1.84–2.0 m/s), respectively.

**Table 4:** Summary of diagnostic accuracy of ultrasound elastography for BA.

**References**	**Technique**	**Cut-off value**	**AUROC**	**Sensitivity (%)**	**Specificity (%)**	**PPV (%)**	**NPV (%)**
Boo et al. (12)	TE	7.7 kPa	NA	80.0	97.8	92.3	93.8
Wang et al. (32)	2D-SWE	8.68 kPa	0.997	97.4	100	100	96.9
Zhou et al. (33)	2D-SWE	10.2 kPa	0.79	81.4	66.7	76	73.5
Wu et al. (34)	TE	7.7 kPa	0.853	80	97	92.31	91.43
Dillman et al. (35)	p-SWE	1.53 m/s	0.81	76.9	78.6	62.5	88.0
	2D-SWE	1.84 m/s	0.89	92.3	78.6	66.7	95.7
Duan et al. (36)	2D-SWE	12.35 kPa	0.937	84.3	89.7	82.7	90.7
Shen et al. (37)	2D-SWE	9.5 kPa	0.771	73.3	70.1	69.2	74.1
Liu et al. (38)	p-SWE	1.77 m/s	0.889	90.9	68.4	69.4	90.5
	2D-SWE	1.92 m/s	0.918	95.5	78.9	78.1	95.7
Sandberg et al. (39)	p-SWE^a^	1.6 m/s	0.8	80	64	81.6	61.5
	2D-SWE	2.0 m/s	0.7	71	67	81.1	53.6
Wang et al. (40)^b^	2D-SWE	7.81 kPa	0.888	87.6	78.5	63.9	93.6
Liu et al. (41)	2D-SWE	7.10 kPa	0.82	81.93	69.86	75.6	77.3

BA, biliary atresia; NA, not available; NPV, negative predictive value; PPV, positive predictive value; p-SWE, point shear wave elastography; TE, transient elastography; 2D-SWE, two-dimensional shear wave elastography.

a Data were obtained using L9 point shear wave elastography (p-SWE).

b Data in the combined training and validation cohort.

**Figure 3:**
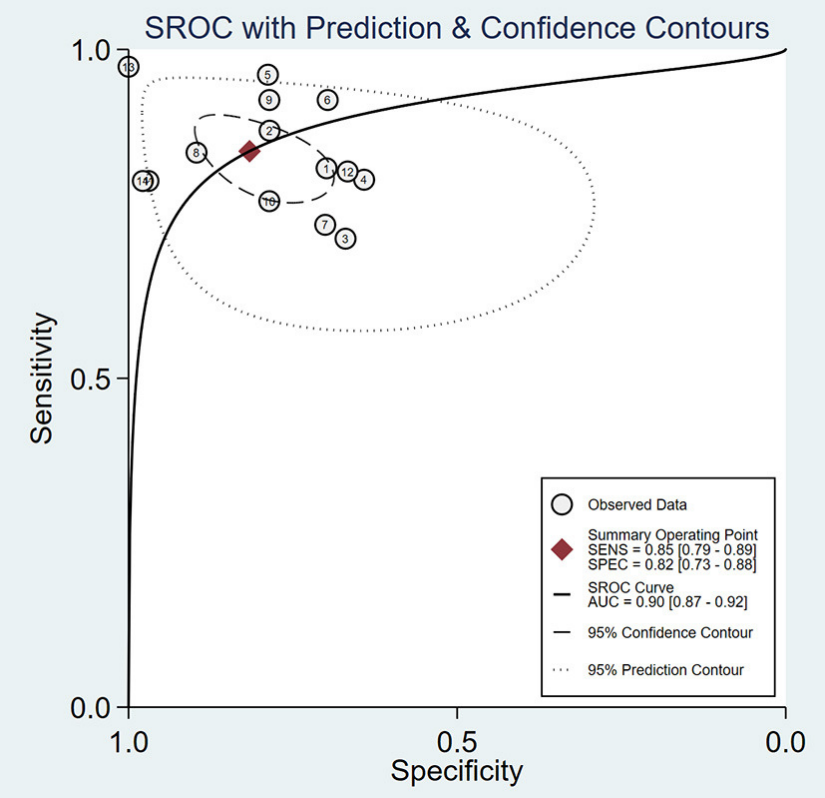
Summary receiver operating characteristic (SROC) curve of the diagnostic performance of ultrasound elastography for the diagnosis of biliary atresia (BA).

**Figure 4:**
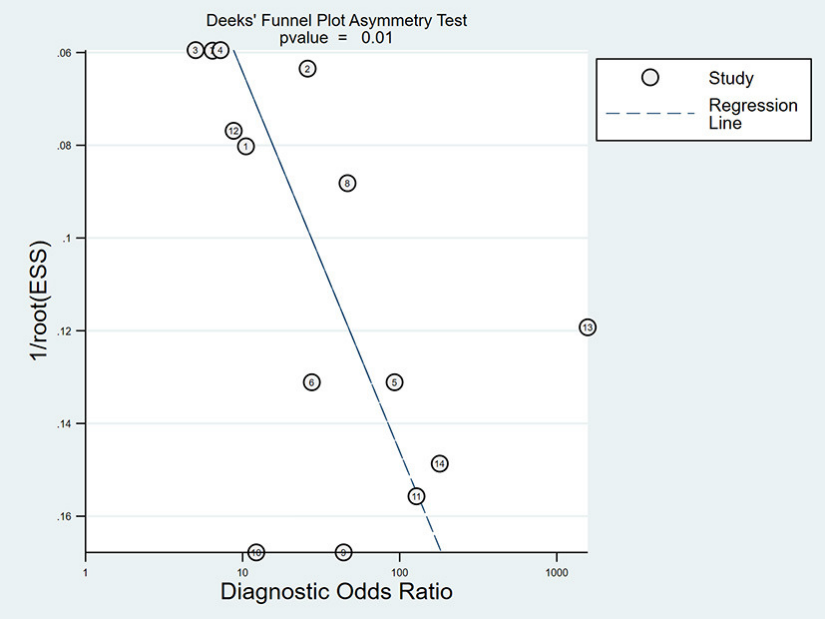
Deeks' funnel plot used to assess publication bias.

As is shown in Figure 3, the summary AUROC value was 0.90 (95% CI: 0.87–0.92) when ultrasound elastography was used for diagnosing BA. The summary DOR value was 24 (95% CI: 11–51). A Deeks' funnel plot asymmetry test was used to evaluate possible publication bias (Figure 4). When ultrasound elastography was used to diagnose BA, a significant publication bias was present (*P* < 0.05).

**Figure 5:**
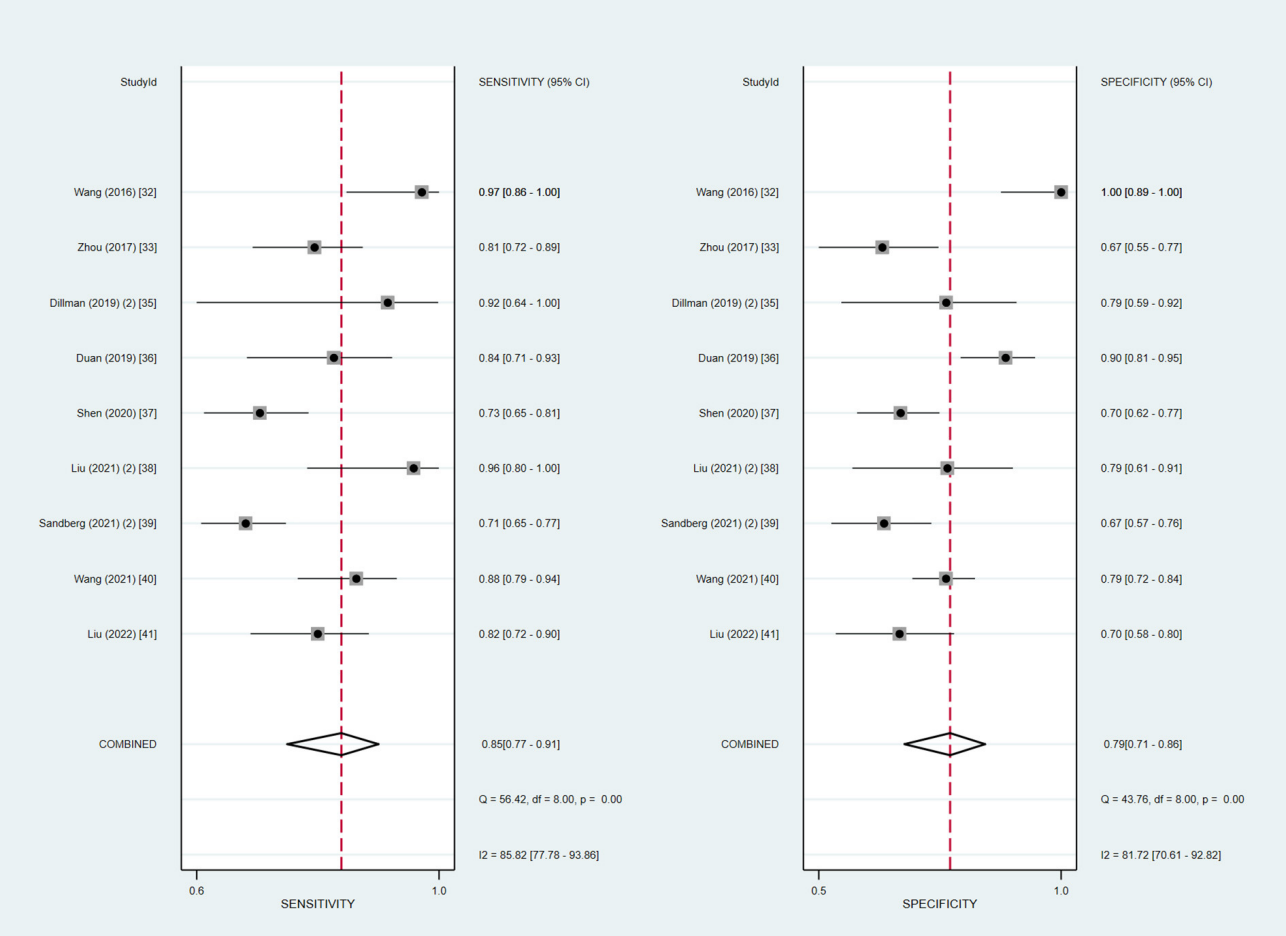
Coupled forest plots of the summary sensitivity and specificity of the liver stiffness measurement in the two-dimensional shear wave elastography (2D-SWE) subgroup.

In addition, we also summarized the relevant studies using the 2D-SWE technique and evaluated the diagnostic accuracy of 2D-SWE in diagnosing BA. In our meta-analysis, a subgroup analysis of the nine studies using 2D-SWE indicated pooled sensitivity and specificity of 85% (95% CI: 77–91%) and 79% (95% CI: 71–86%) with the *I*^2^ value of 85.82 and 81.72%, respectively (Figure 5). The summary AUROC value of 2D-SWE was 0.89 (95% CI: 0.86–0.92), for diagnosing BA. Moreover, the summary DOR value was 22 (95% CI: 9–57).

### Heterogeneity and subgroup analysis

The Spearman correlation coefficient was −0.275 (*P* = 0.342), confirming that the threshold effect was not significant in ultrasound elastography studies and, therefore, a non-threshold heterogeneity was examined by *I*^2^ statistics. The inconsistency index *I*^2^ statistic revealed substantial heterogeneity with regard to the summary sensitivity and specificity among studies (*I*^2^ = 82.90 and 84.33%, respectively) (Figure 2).

To explore the origin of the heterogeneity, we further performed subgroup analysis and meta-regression analysis. The meta-regression analysis showed that publication year, method of processing measurement, ultrasound elastography systems, and ultrasound elastography techniques could be the reasons of the heterogeneity. The subgroup analysis based on ultrasound elastography technique showed that 2D-SWE had higher sensitivity (85 vs. 81%) and AUROC (0.89 vs. 0.82) but lower specificity (79 vs. 87%) than other ultrasound elastography techniques in diagnosing BA. More detailed information regarding the subgroup analysis is described in Table 5.

**Table 5:** Summary results of subgroup analysis for ultrasound elastography in the diagnosis of BA.

**Subgroup**	**Summary sensitivity (95% CI, %)**	**Summary specificity (95% CI, %)**	**Summary LR+ (95% CI)**	**Summary LR- (95% CI)**	**Summary AUROC (95% CI)**	**Summary DOR (95% CI)**	***I*** **^2^ (%)^c^**
**Publication year**							
≥2020^a^	83 (76–88)	75 (68–81)	3.4 (2.4–4.7)	0.23 (0.15–0.35)	0.86 (0.83–0.89)	15 (7–31)	80.62, 75.97
<2020	87 (79–92)	89 (73–96)	8.2 (2.9–23.1)	0.15 (0.08–0.25)	0.92 (0.89–0.94)	56 (13–246)	61.08, 86.49
**Method of processing measurement**							
Mean	88 (77–94)	83 (68–92)	5.1 (2.5–10.5)	0.15 (0.07–0.31)	0.92 (0.90–0.94)	34 (9–138)	86.05, 88.43
Median	81 (75–87)	81 (69–89)	4.3 (2.5–7.4)	0.23 (0.16–0.33)	0.86 (0.83–0.89)	19 (8–43)	80.01, 83.03
**US elastography systems**							
Aixplorer^b^	82 (78–85)	75 (71–78)	3.0 (2.2–4.1)	0.23 (0.14–0.37)	0.93	14 (6–31)	76.7, 83.8
Others	83 (76–88)	83 (72–90)	4.8 (2.9–8.2)	0.21 (0.14–0.31)	0.89 (0.85–0.91)	24 (10–53)	83.57, 85.47
**US elastography techniques**							
2D-SWE	85 (77–91)	79 (71–86)	4.1 (2.7–6.3)	0.18 (0.11–0.31)	0.89 (0.86–0.92)	22 (9–57)	85.82, 81.72
Others	81 (73–87)	87 (66–95)	6.0 (2.1–16.9)	0.22 (0.15–0.32)	0.82 (0.78–0.85)	27 (8–96)	76.46, 89.48

AUROC, area under the ROC curve; BA, biliary atresia; CI, confidence interval; DOR, diagnostic odds ratio; LR+, positive likelihood ratio; LR-, negative likelihood ratio; 2D-SWE, two-dimensional shear wave elastography; US, ultrasound.

a Median of all included studies.

b SuperSonic Imagine.

c Estimation of the inconsistency index *I*^2^ statistic for sensitivity and specificity.

## Discussion

Measuring liver stiffness with ultrasound elastography in cholestatic infants might be helpful in the differential diagnosis of BA (2, 34). To this end, based on data acquired from published studies, we conducted the present meta-analysis to provide evidence-based insight regarding the diagnostic performance of ultrasound elastography for BA.

In this meta-analysis, we identified 14 studies from 11 articles with 1,797 subjects (including 774 BA patients, 850 non-BA patients, and 173 controls) for evaluation, and found that ultrasound elastography provided a summary sensitivity of 85% (95% CI: 79–89%), specificity of 82% (95% CI: 73–88%), and AUROC of 0.90 (95% CI: 0.87–0.92). Our findings indicated that liver stiffness measurement by ultrasound elastography had a good diagnostic accuracy for the diagnosis of BA.

BA is a challenging liver disease in infancy (12). It remains the leading indication for pediatric liver transplantation throughout the world, despite surgical treatment (8, 42). Of note, time-to-treatment is a critical factor in determining outcome (43). For timely diagnosis of BA, therefore, a non-invasive and accurate diagnostic tool is required (12). Several quantitative ultrasound elastography techniques such as TE, p-SWE and 2D-SWE have been widely used to evaluate pediatric liver diseases such as fibrosis and BA by measuring liver stiffness (20, 44). In recent years, a good diagnostic performance for ultrasound elastography has been extensively reported in pediatric patients, especially with liver diseases. Of interest, in a recent meta-analysis of 12 studies including 550 pediatric patients, the summary sensitivity and specificity of ultrasound shear wave elastography for predicting liver significant fibrosis were 81% (95% CI: 71–88%) and 91% (95% CI: 83–96%), respectively (45). Hwang et al. (46) in a meta-analysis reported a summary sensitivity of 95% (95% CI: 74–99%) and a specificity of 90% (95% CI: 81–95%) for TE for the evaluation of significant liver fibrosis in children with an AUROC of 0.96 (95% CI: 0.94–0.98). Another previous meta-analysis performed by Kim et al. (21) found that, for evaluation of portal hypertension in children, the summary sensitivity and specificity of ultrasound elastography were 90% (95% CI: 83%–94%) and 79% (95% CI: 73–84%), respectively, and the summary AUROC was 0.92 (95% CI: 0.90–0.94). This suggests that ultrasound elastography is a promising tool for assessment of liver diseases in the pediatric population (44).

Nevertheless, in a real clinical practice, some factors specific to pediatric patients that may affect liver stiffness measurement with ultrasound elastography need to be considered, such as age, probe choice, a small and thin body size, and ability to lay still and cooperate (20, 25, 45, 46). For example, even under ideal conditions, the success rate of TE for the measurement of liver stiffness in children younger than 24 months was lower (25).

This meta-analysis demonstrated that, for ultrasound elastography, the summary sensitivity and specificity for the diagnosis of BA were 85% (95% CI: 79–89%) and 82% (95% CI: 73–88%), respectively. Moreover, a subgroup analysis of nine 2D-SWE studies was also performed and was shown to have similar diagnostic performance. The summary sensitivity and specificity of liver stiffness measured by 2D-SWE for diagnosing BA were 85% (95% CI: 77%–91%) and 79% (95% CI: 71–86%), respectively. The summary AUROC was 0.89 (95% CI: 0.86–0.92). Therefore, ultrasound elastography, as a promising novel imaging modality, could aid in accurate diagnosis of BA.

It is worth noting that, in several previous studies (33, 36, 39), the diagnostic performance of ultrasound elastography for BA was reported to be inferior to that of conventional ultrasound. In Zhou's study (33), for identifying BA, the performance of liver stiffness measurement was not exceed that of gray-scale ultrasound findings, including fibrotic cord thickness and gallbladder classification (AUROC: 0.790 vs. 0.868–0.922). In the original study of Sandberg et al. (39), triangular cord sign was reported to be the strongest predictor for BA among the individual gray-scale imaging findings (sensitivity: 88%; specificity: 96%), which was superior to the p-SWE (sensitivity: 80%; specificity: 64%) and 2D-SWE (sensitivity: 71%; specificity: 67%). Nevertheless, the use of gray-scale ultrasound findings including triangular cord sign to identify BA requires an analyst with expertise (10, 17). It is somewhat subjective and operator dependent. Notably, a previous study found that in up to 83% of BA patients within 30 days and 44% of BA patients over 30 days, the triangular cord sign was absent (47).

Due to some factors such as different types of ultrasound elastography systems and techniques, and different age groups and serum biochemical index levels in infants with cholestasis, the optimal cut-off values proposed for BA diagnosis varied across the studies included in our meta-analysis. It should be noted that different vendors and different probes could be expected to have different stiffness values (44). Hence, it is important in clinical practice to recognize the differences between the various ultrasound elastography systems and techniques used to measure liver stiffness (19, 20).

A major strength of the present meta-analysis is that we comprehensively investigated the diagnostic performance of liver stiffness, measured by different ultrasound elastography systems and techniques including TE, p-SWE and 2D-SWE. We also conducted a subgroup analysis and assessed the performance of 2D-SWE for the diagnosis of BA. In contrast to TE and p-SWE, 2D-SWE is a relatively novel ultrasound elastography technique (19, 48). Finally, through subgroup analysis and meta-regression analysis, we could explore the origin of heterogeneity.

Some notable limitations of this study should be acknowledged. First, substantial heterogeneity was observed across the studies included in this meta-analysis. Also, a significant publication bias was also present among studies. This might be attributable to several reasons, such as the types of ultrasound elastography technique from different vendors, study population, a variable distribution of severity of the disease, and study design, etc. Additionally, most included studies were small in size. Thus, any interpretation of the results of our present meta-analysis should be mindful of the heterogeneity and publication bias. Second, due to the limited number of articles, our meta-analysis included studies that used different ultrasound elastography systems and techniques. Nevertheless, we conducted subgroup analysis based on ultrasound elastography system and technique. Further studies are required to investigate the diagnostic performance of specific ultrasound elastography systems/techniques for BA. Third, we only included studies published in English, leading to a linguistic bias. In addition, most of the included studies were from China. Due to this, caution is necessary in interpreting our findings.

In conclusion, ultrasound elastography exhibits good performance in the diagnosis of BA and can be served as a non-invasive tool to facilitate the differential diagnosis of BA from other neonatal cholestasis.

## Data availability statement

The original contributions presented in the study are included in the article/Supplementary material, further inquiries can be directed to the corresponding author/s.

## Author contributions

BD, XY, and GL contributed to the study design and literature search. BD, ZW, XY, and HW completed the data analysis. XY and HW generated and improved the figures and tables. BD completed the manuscript. BD, ZW, and GL proofread the manuscript. All authors contributed to the article and approved the submitted version.

## Funding

This work was supported by Collaborative Innovation Center for Maternal and Infant Health Service Application Technology from Quanzhou Medical College (grant no. XJM1802).

## Conflict of interest

The authors declare that the research was conducted in the absence of any commercial or financial relationships that could be construed as a potential conflict of interest.

## Publisher's note

All claims expressed in this article are solely those of the authors and do not necessarily represent those of their affiliated organizations, or those of the publisher, the editors and the reviewers. Any product that may be evaluated in this article, or claim that may be made by its manufacturer, is not guaranteed or endorsed by the publisher.
