# Supplementary figures and images for: The diagnostic performance of ultrasound elastography for biliary atresia: A meta-analysis
Source: Front Public Health. 2022 Oct 26;10:973125. doi: 10.3389/fpubh.2022.973125 (PMC9643747; doi:10.3389/fpubh.2022.973125)

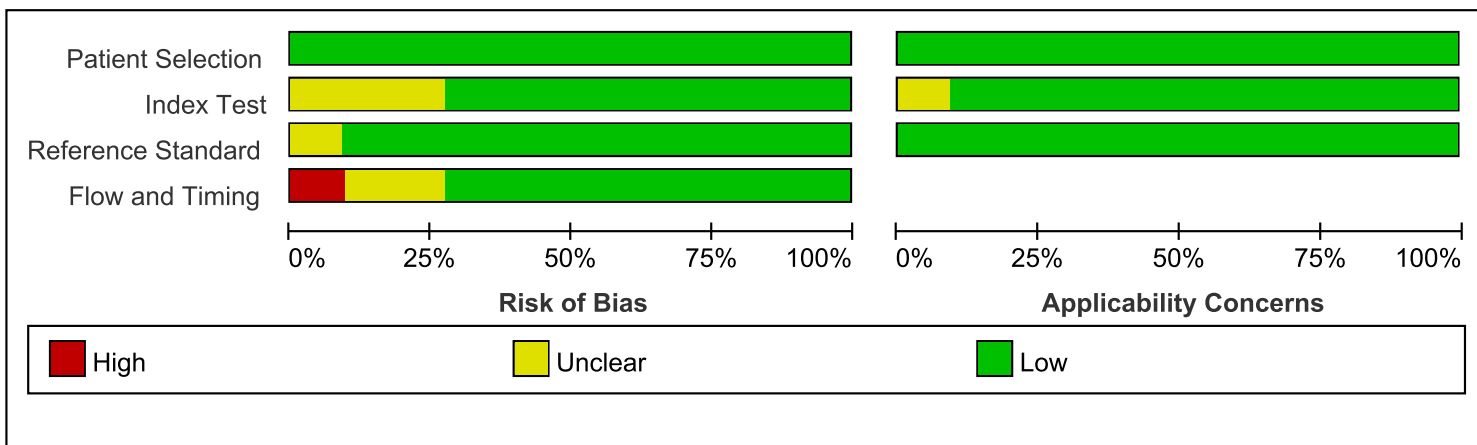

Supplement: Supplementary file 1 [file Data_Sheet_1.PDF]
